# Impact of anthropogenic accumulation on phytoplankton community and harmful algal bloom in temporarily open/closed estuary

**DOI:** 10.1038/s41598-023-47779-1

**Published:** 2023-12-27

**Authors:** Ponnusamy Sathish Kumar, Dharani Gopal, Dilip Kumar Jha, Krupa Ratnam, Santhanakumar Jayapal, Vikas Pandey, Venkatnarayanan Srinivas, Arthur James Rathinam

**Affiliations:** 1 Ocean Science and Technology for Islands, National Institute of Ocean Technology (NIOT), Ministry of Earth Sciences, Pallikaranai, Chennai, India; 2 Department of Marine Science, Bharathidasan University, Tiruchirappalli, Tamil Nadu India

**Keywords:** Biogeochemistry, Environmental sciences

## Abstract

Spatio-temporal variation in phytoplankton community dynamics in a temporarily open/closed Swarnamukhi river estuary (SRE), located on the South East coast of India was investigated and correlated to that of the adjacent coastal waters. Understanding the seasonal variability of the phytoplankton community and influencing factors are essential to predicting their impact on fisheries as the river and coastal region serve as the main source of income for the local fishing communities. Downstream before the river meets the sea, an arm of the Buckingham Canal (BC), carrying anthropogenic inputs empties into the Swarnamukhi River (SR1). The impact of anthropogenic effects on the phytoplankton community at BC was compared to other estuarine stations SR2 (upstream), SR1 (downstream), SRM (river mouth) and coastal station (CS). In BC station, harmful algal blooms (HABs) of *Chaetoceros decipiens* (2940 × 10^3^ cells L^−1^) and *Oscillatoria* sp. (1619 × 10^3^ cells L^−1^) were found during the southwest monsoon and winter monsoon, respectively. These HABs can be linked to the anthropogenic input of increased nutrients and trace metals. The HABs of *Oscillatoria* sp. were shown to be induced by elevated concentrations of nitrate (10.18 µM) and Ni (3.0 ppm) compared to ambient, while the HABs of C. *decipiens* were caused by elevated concentrations of silicate (50.35 µM), nitrite (2.1 µM), and phosphate (4.37 µM). Elevated nutrients and metal concentration from the aquaculture farms, and other anthropogenic inputs could be one of the prime reasons for the recorded bloom events at BC station. During this period, observed bloom species density was found low at other estuarine stations and absent at CS. The formation of bloom events during the closure of the river mouth could be a major threat to the coastal ecosystem when it opens. During the *Osillatoria* sp. bloom, both the Cu and Ni levels were higher at BC. The elevated concentration of nutrients and metals could potentially affect the coastal ecosystem and in turn fisheries sector in the tropical coastal ecosystem.

## Introduction

Phytoplankton play a crucial role in the marine food web. Composition and the spatio-temporal variability of phytoplankton community reflect both short- and long-term environmental changes in the aquatic ecosystem^1^. Several environmental factors, including temperature, nutrient availability, and river runoff^2,3^ determine the growth, distribution, and composition of phytoplankton in the coastal and marine environment. Estuarine systems, which are influenced by both natural fluctuation (tidal and seasonal) and anthropogenic activities (land and river-runoff) are complex and dynamic environmental niches in terms of their hydrological conditions & community composition. The land runoffs usually increase the load of pathogens, nutrients, trace metals, detergents, and pesticides in the receiving water body^4^. In closed estuarine systems increased supply of nutrients results in eutrophication that could trigger harmful algal blooms (HABs) which release toxic secondary metabolites and alter the ecosystem. The occurrence of HABs is increasing in frequency and intensity around the world^5–7^. The occurrence and persistence of algal blooms depends upon a combination of interaction with physical, chemical, and biological factors^8,9^.

On the other hand, pollutants brought by rivers and land runoff are considered major threats to marine ecosystems and might alter food-web dynamics in coastal environments^4,10^. Trace metals enter the estuarine system through natural processes and various anthropogenic activities such as mineral weathering, mining, fossil fuel combustion, industrial discharges, urban development, sewage and agricultural discharge^11,12^. Some trace metals are essential for phytoplankton growth and survival, however, their concentration beyond the permissible level will harm marine ecosystem and seafood safety through persistent bio-accumulation^1,11^. Therefore, both natural and anthropogenic inputs play an important role in controlling the physicochemical and biological factors of an estuarine ecosystem.

Open-closed estuaries are the most complex and important aquatic ecosystems, which differ from other estuaries, in that mixing does not occur during the closure of the river mouth^13^. Swarnamukhi River Estuary is a temporarily open/closed estuarine system, situated along the Pamanji coastal village of Nellore District, Andhra Pradesh, on the southeast coast of India. River mouth closure and the connection between the river and sea is mostly interrupted for 3–5 months during the late summer monsoon to early north-east monsoon (June–October) due to the massive amount of sand deposition. The duration of mouth closure depends on the precipitation during that particular period^14,15^, mostly the river mouth opens during the NEM due to high rainfall. It is of interest to study the phytoplankton community dynamics and the factors influencing them in such temporarily open/closed estuaries. Bloom incidents occurring in open/closed estuaries have not been well-documented^16,17^. Several studies have addressed the phytoplankton community structure in relation to physico-chemical parameters^18–20^, however, there are few studies which have investigated the levels of heavy metals and their role in phytoplankton community structure^21^. In view of these lacunae the present study has been carried out in the Swarnamukhi River Estuarine system which offers a unique habitat to investigate phytoplankton community structure. Objectives of the present study are (1) to investigate the spatio-temporal changes in hydrographical parameters and heavy metals concentration, (2) to investigate the spatio-temporal variations of phytoplankton community and their response to changing environmental variables, and (3) to investigate the influence of heavy metals and other environmental variables on harmful algal blooms.

## Materials and methods

**Figure 1:**
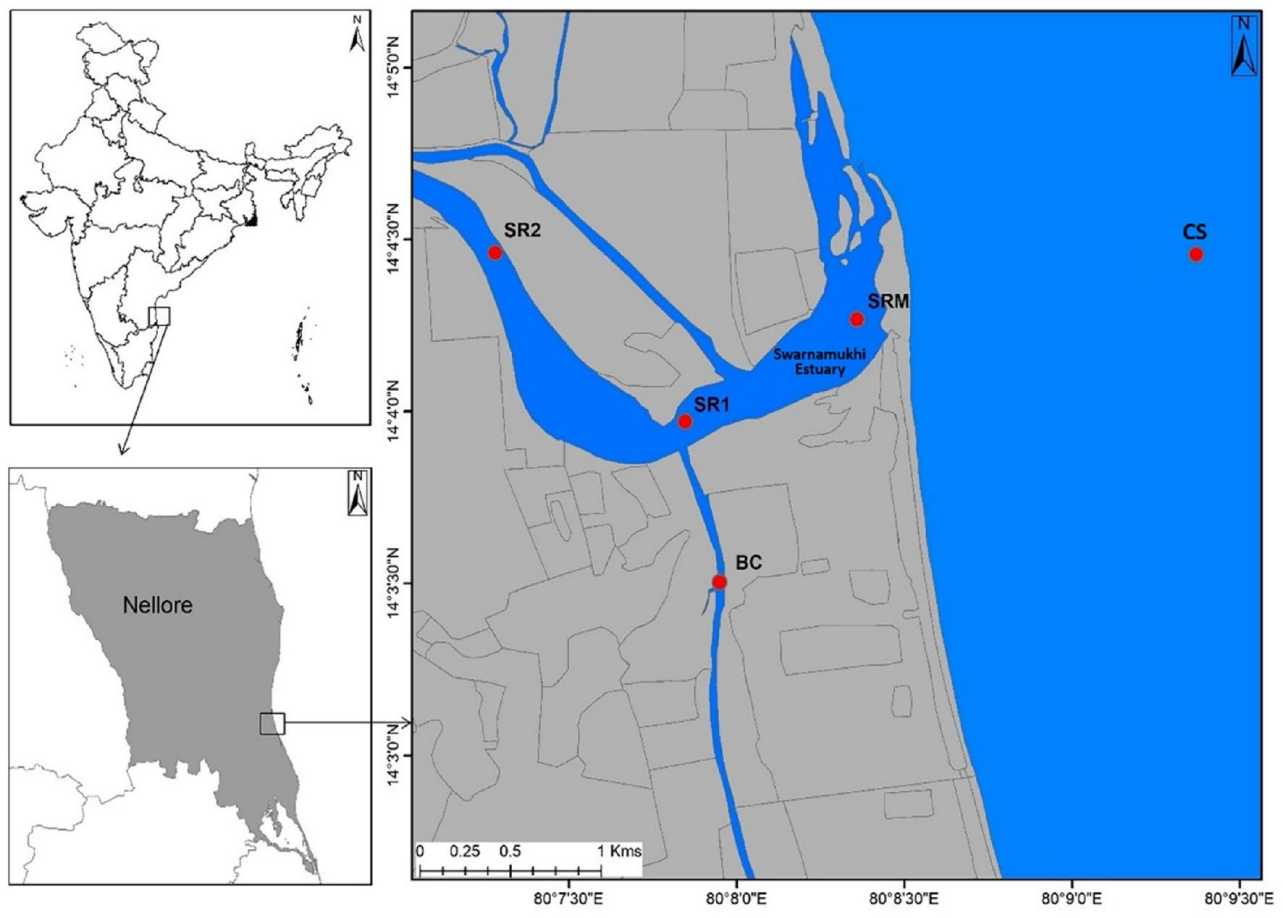
Study locations along the Swarnamukhi River Estuary, southeast coast of India. (Study area map created by ArcGIS ver. 10.1 software, https://www.esri.com/en-us/home).

Sampling was carried out in the temporarily open-closed Swarnamukhi river estuary (SRE) and adjacent coastal waters of Pamanji, Nellore, Southeast coast of India. Duplicate samples were collected for 2 years (2018 and 2019) during four different seasons, i.e., winter monsoon (WM; Jan–Mar), summer monsoon (SM; Apr–May), southwest monsoon (SWM; Jun–Sep), and northeast monsoon (NEM; Oct–Dec). Five sampling stations covering, Coastal Station (CS), Swarnamuki River Estuary (SRM: river mouth, SR1: river downstream, and SR2: river upstream), and Buckingham Canal (BC) were selected (Fig. 1).

Monthly mean rainfall data for the sampling period was collected from Indian Meteorological Department (IMD). Water temperature (WT), salinity, and pH were measured using an in situ calibrated portable multi-parameter water quality instrument (Hana HI 9829). Water samples were collected from the surface (~ 0.5 m) using a 5 L Niskin sampler (Hydrobios-Kiel) for measuring dissolved oxygen (DO), biochemical oxygen demand (BOD), total suspended solids (TSS), chlorophyll-*a* (Chl-*a*), nutrients, and trace metals. The DO and BOD were measured by modified Winkler’s method^22^. To determine TSS, a measured volume of sample was filtered through pre-dried and pre-weighed (0.45 mm, Millipore GF/C) filter paper and washed with Milli-Q water to remove salt contents^23^. Water samples collected for dissolved nutrient analysis were filtered by GF/F filter paper for removing the particulate matter and the filtrate was frozen at − 20 °C until further analysis. All nutrients (Ammonium (NH_4_^+^), nitrite (NO_2_^−^), nitrate (NO_3_^−^), phosphate (PO_4_^3–^), silicate (SiO_4_^2–^), total nitrogen (TN), and total phosphorus (TP)) were analyzed using standard spectrophotometric procedures^24^. The detection limits of NH_4_^+^, NO_2_^−^, NO_3_^−^, PO_4_^3–^, SiO_4_^2–^, TN and TP were ± 0.02, ± 0.01, ± 0.02, ± 0.01, ± 0.02, ± 0.02 and ± 0.01 μM, respectively. For trace metal analysis, unfiltered water samples were collected in acid-cleaned polypropylene bottles and immediately acidified to pH 2–3 using supra-pure HNO_3_ and kept at 4 °C until analysis. For digestion, 5 mL of water sample was added with 5 mL of concentrated HNO_3_ and digested in microwave. The digested samples were transferred to centrifuge tubes, and made the volume up to 50 mL by adding Milli-Q water^25^. Trace metals concentrations were determined by using Inductively Coupled Plasma Mass Spectrometry (ICP-MS; Agilent-7500). The digestion and analytical procedures were carried out according to the method given by the US Environmental Protection Agency^26^. The samples, certified reference materials (CRM), and duplicate analytical blanks were examined for any potential contamination as per the protocol followed by Jha et al. ^27,28^. The reference value (seawater), analyzed mean and standard deviation of the elemental values obtained for the NIST CRM QC3163 (seawater), is adopted from Jha et al. ^27^. By comparing the analyzed value to the CRM value, the precision and correctness of the analysis were confirmed. The metal concentration in water sample was represented as ppb.

For Chl-*a* analysis, a known volume of water sample was filtered through Whatman GF/F filter and preserved at − 20 °C until analysis. Chl-*a* in the filter was extracted with 90% acetone at 4 °C in the dark for 24 h and analysed spectrofluorometrically^29^. For phytoplankton analysis, water sample was collected 5 L for coastal station, and 1 L for estuarine stations, and preserved with 4% Lugol’s iodine solution. Utermohl’s sedimentation method was used to count the phytoplankton under microscope (NIKON Eclipse E100), and identification was carried out with the standard identification keys^30–32^.

Shannon–Wiener’s diversity index (H′), Margalef’s species richness (d) and Pielou’s evenness (J′) were determined by using PRIMER-7. The statistical significance of diversity indices and environmental parameters were assessed using one-way analysis of variance (ANOVA) on XLSTAT software. Pearson correlation analysis was performed for environmental variables with phytoplankton biomass and abundance by XLSTAT statistical software. Principal component analysis (PCA) was used to analyse the spatio-temporal distributions of the environmental parameters. Redundancy analysis (RDA) was used to examine the relationship between the dominant phytoplankton species and environmental variables. PCA and RDA were employed by using CANACO statistical software.

## Results and discussion

### Spatio-temporal variation of hydrographic parameters and heavy metals

**Figure 2:**
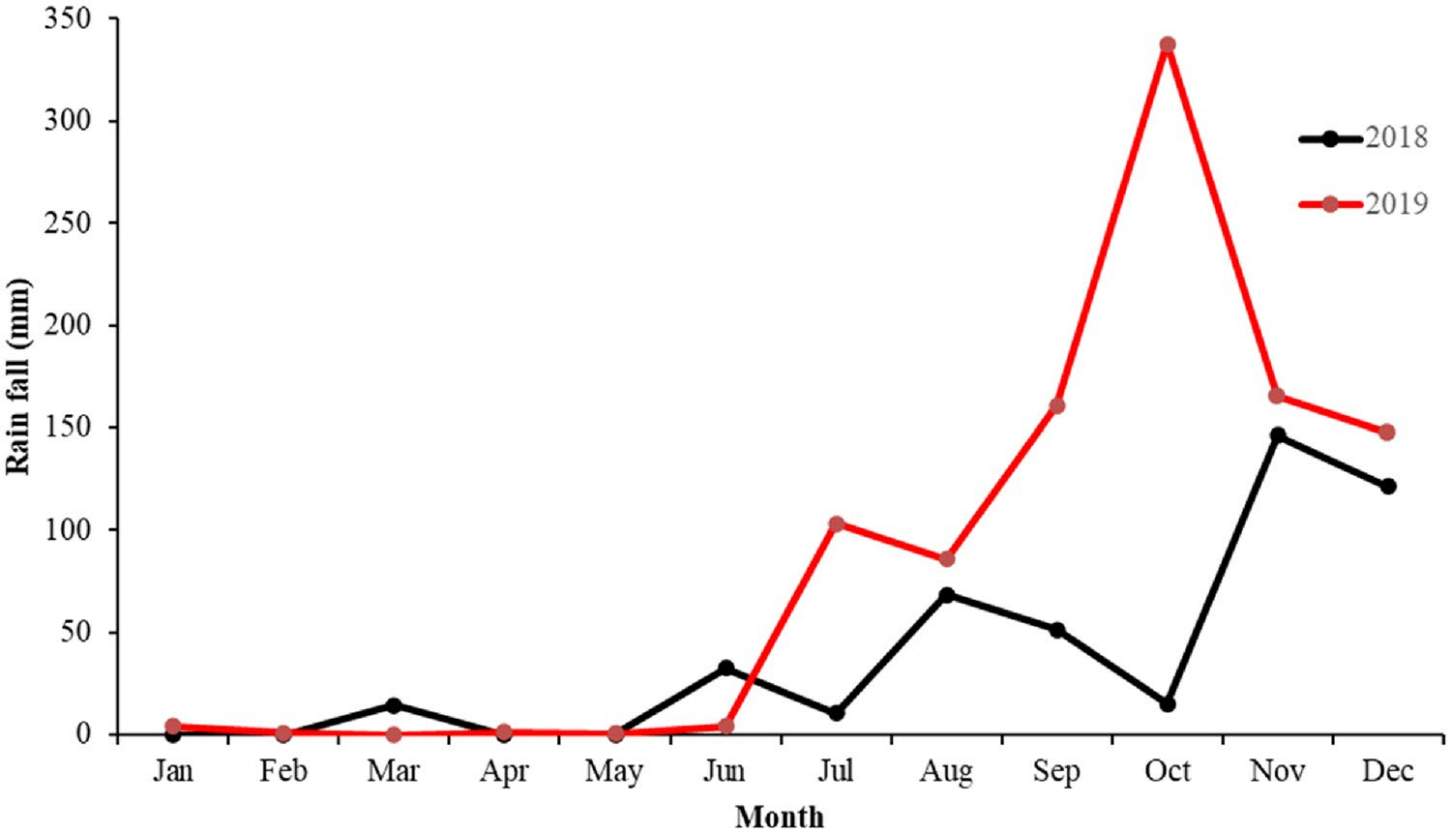
Mean monthly rainfall in the Swarnamukhi River Estuary, southeast coast of India.

In the present study, maximum rainfall was recorded during the NEM followed by SWM (Fig. 2). High rainfall (337.7 mm) was recorded during the NEM (Oct 2019). The average rainfall in the Nellore coastal region ranges from 762 to 1270 mm^33^, which was about 50% lesser during 2018 in the present study area. Most of the environmental parameters showed a significant spatial variability between the seasons (Table 1). The salinity was comparatively high during the SM (33.76 PSU) than the WM (27.71 PSU). However, salinity did not show a significant seasonal variation in the SRE, which could be due to the low rainfall during the study period. Further, limited freshwater discharge and presence of reservoirs/dams at upstream result in low runoff which could be attributed to high salinity observed, unlike other estuarine systems^34^. In the present investigation, comparatively higher salinity was recorded in the CS, whereas it was low in BC and SR2 stations. The WT ranged from 26.6 to 32.9 °C with the higher WT recorded during the SM in SR2 station and lower during the WM at CS. However, during the NEM, SRM station recorded high WT, which might be due to the closure of river mouth and lack of water exchange^15^. TSS ranged from 5.3 to 20.8 mg L^−1^, with the mean concentration being higher during the SWM and lower during the WM. TSS levels in BC and SRM were consistently high throughout the year (Table S1), attributed to continuous discharge from shrimp farms, and sediment disturbance due to runoff & mixing at the convergence zone of sea and river water, respectively^14,27^. The low DO at BC during all the seasons, could be due to the organic enrichment from aquaculture effluents discharge^35^. Dissolved inorganic nutrient levels were fluctuating among the seasons (Table 1). The NO_3_^−^ concentration was higher during the SWM (11.23 µM) and lower during the SM (0.31 µM). A similar trend was observed for NO_2_^−^ and NH_4_^+^. Spatially, increased NO_2_^−^ and ${\text{NO}}_{{3}}^{ - }$ concentration was observed in the BC and SR2 might be due to the anthropogenic input in the region^19,36^. Higher NH_4_^+^ concentration was recorded in SRM during the SWM could be attributed to non-mixing of oceanic water due to closure of the SRE mouth^14^. During all the seasons, most of the nutrient concentration were close to optimal level in the CS, and increased gradually from downstream to upstream and BC, which could be attributed to high land runoff into the river. Estuarine-coastal ecosystems are usually characterized with increased nutrient inputs from untreated domestic sewage, industrial waste, and agricultural runoff^4,7^. Spatially, the SiO_4_^2–^and PO_4_^3–^concentrations (Table S1) were always 2–3 times higher at BC than the other stations. The mean SiO_4_^2–^and PO_4_^3–^concentrations were high during the SWM. Increased PO_4_^3–^ concentration during the SWM might be due to the usage of phosphorus compounds for the aquaculture and agriculture operations^37^.

**Table 1:** Seasonal variation of environmental variables in the Swarnamukhi River Estuary, southeast coast of India.

Parameters	WM	SM	SWM	NEM
WT (°C)	26.64–28.75	30.67–32.93	30.0–30.88	29.0–30.0
	(27.75 ± 0.35)	(32.13 ± 0.44)	(30.53 ± 0.18)	(29.32 ± 0.21)
pH	8.14–8.37	8.24–8.40	8.06–8.39	8.13–8.33
	(8.26 ± 0.04)	(8.34 ± 0.03)	(8.24 ± 0.05)	(8.23 ± 0.03)
Salinity (PSU)	27.71–31.40	32.46–33.76	28.11–31.96	28.64–31.75
	(29.0 ± 0.66)	(33.14 ± 0.22)	(29.66 ± 0.78)	(29.70 ± 0.60)
TSS (mg L^−1^)	5.87–17.40	7.10–19.50	12.0–18.12	5.30–20.80
	(9.37 ± 2.08)	(14.29 ± 2.05)	(15.39 ± 1.0)	(13.52 ± 2.53)
DO (mg L^−1^)	3.01–5.12	2.28–4.63	3.58–4.23	4.15–5.20
	(3.80 ± 0.37)	(3.71 ± 0.39)	(3.90 ± 0.12)	(4.80 ± 0.22)
BOD (mg L^−1^)	0.0–3.10	1.71–3.90	1.41–4.23	2.09–3.22
	(1.28 ± 0.62)	(2.55 ± 0.39)	(3.42 ± 0.52)	(2.90 ± 0.21)
NH_4_^+^ (µM)	0.97–4.22	0.1–8.33	1.0–9.33	0.40–7.25
	(1.85 ± 0.61)	(1.88 ± 1.63)	(6.13 ± 1.39)	(2.54 ± 1.24)
NO_3_^−^ (µM)	2.40–10.18	0.31–2.29	1.16–11.23	1.57–9.96
	(5.50 ± 1.43)	(0.86 ± 0.36)	(5.75 ± 1.90)	(4.45 ± 1.49)
NO_2_^−^ (µM)	0.13–0.37	0.12–1.60	0.17–2.10	0.14–1.65
	(0.29 ± 0.04)	(0.49 ± 0.28)	(1.02 ± 0.37)	(0.55 ± 0.28)
PO_4_^3–^ (µM)	0.10–1.01	0.25–0.88	0.43–4.37	0.08–0.54
	(0.41 ± 0.16)	(0.44 ± 0.12)	(1.63 ± 0.70)	(0.32 ± 0.09)
SiO_4_^2–^ (µM)	9.87–44.87	8.58–35.32	8.69–50.35	16.86–58.74
	(25.69 ± 6.92)	(19.36 ± 4.84)	(31.13 ± 8.25)	(30.04 ± 7.37)
TN (µM)	4.44–26.86	11.35–37.12	6.55–29.69	4.88–20.16
	(12.08 ± 4.42)	(21.88 ± 5.13)	(19.54 ± 4.44)	(9.47 ± 2.76)
TP (µM)	0.70–3.22	0.77–3.96	1.93–6.65	1.28–2.50
	(1.97 ± 0.45)	(2.60 ± 0.54)	(4.28 ± 0.92)	(1.77 ± 0.24)
Chl-*a* (mg m^−3^)	0.24–4.66	0.17–4.53	0.18–17.18	0.80–7.95
	(1.54 ± 0.84)	(1.81 ± 0.83)	(5.58 ± 3.19)	(2.78 ± 1.32)
Phytoplankton density (× 10^3^ Cells L^−1^)	7.5–1682.8	16.0–382.2	18–3001.8	5.70–295.4
	(391.6 ± 325.7)	(103.3 ± 70.0)	(666.2 ± 584.3)	(77.4 ± 55.2)

Values in the open and parentheses represent the minimum–maximum and mean values with ± standard error, respectively.

Result of heavy metal levels measured in the present study is given in Table 2. The mean concentrations of heavy metals in seawater decreased in the following order in the present study area: Fe (104.6 ± 26.4 ppb) > Al (61.0 ± 12.7) > Mn (35.6 ± 14.3 ppb) > Zn (8.5 ± 1.4 ppb) > Cu (1.5 ± 0.2 ppb) > Cr (1.5 ± 0.4 ppb) > Ni (1.4 ± 0.2 ppb) > Pb (0.7 ± 0.1 ppb) > Co (0.3 ± 0.04 ppb) > Hg (BDL). Most of the trace metal concentrations were higher during the SM followed by SWM in the river upstream and BC stations. In the CS, Fe and Cr showed elevated levels during the SM, and Mn during the WM. The transportation of iron ores from Krishnapatnam port may be a potential source for the elevated levels of Fe (483.73 ppb) observed in the coastal water^38^. The high concentration of Cu at BC station during the WM corroborated with an earlier report and was attributed to the use of biocides in ships and boats, as well as organic inputs from agriculture and industries^27,38^. Ni found to be above water quality index^39,40^ (Table 2) at BC station during the SWM followed by the WM and NEM. Prime source of Ni might be due to wastewater effluent^41^.

**Table 2:** Seasonal variation of trace metal concentration in the Swarnamukhi River Estuary, southeast coast of India.

Parameters	WQGs (ppb)	WM	SM	SWM	NEM
Fe (ppb)	100^a^	86.47–107.6	118.42–483.73	24.96–68.57	16.54–55.98
		(97.04 ± 6.68)	(235.78 ± 68.11)	(44.99 ± 7.18)	(36.22 ± 7.07)
Al (ppb)	200^b^	24.71–129.96	72.16–191.83	0.99 -34.02	9.31–32.47
		(79.63 ± 23.27)	(121.74 ± 20.94)	(16.29 ± 5.35)	(17.8 ± 4.73)
Co (ppb)	5^a^	0.08–0.33	0.31–0.50	BDL	BDL
		(0.20 ± 0.05)	(0.43 ± 0.04)		
Cr (ppb)	10^b^	0.09–1.46	3.70–5.49	0.11–0.84	0.03–0.19
		(0.90 ± 0.23)	(4.52 ± 0.31)	(0.32 ± 0.13)	(0.08 ± 0.03)
Zn (ppb)		1.90–8.05	8.40–25.52	7.06–12.53	1.42–4.20
	30^b^	(4.43 ± 1.27)	(16.37 ± 2.74)	(10.35 ± 1.0)	(2.91 ± 0.48)
Cu (ppb)	10^b^	0.61–4.24	0.87–2.56	1.10–2.73	0.65–1.49
		(1.65–0.78)	(1.43 ± 0.29)	(2.01 ± 0.32)	(1.04 ± 0.16)
Mn (ppb)	100^a^	6.05–281.67	10.63–84.23	0.21–35.37	0.40–8.86
		(73.77 ± 52.36)	(42.70 ± 14.37)	(14.82 ± 6.22)	(4.90 ± 1.55)
Ni (ppb)	2^b^	0.40–3.0	1.06–1.75	0.96–3.13	0.32–2.55
		(1.48 ± 0.55)	(1.47 ± 0.13)	(1.78 ± 0.37)	(0.99 ± 0.40)
Pb (ppb)	3^b^	0.09–0.68	BDL	0.77–1.08	BDL
		(0.48 ± 0.15)		(0.93 ± 0.10)	

Values in the open and parentheses represent the minimum–maximum and mean values with ± standard error, respectively. (BDL, below detection limit; WQGs, water quality guidelines.

^a^Shanmugam et al.^40^

^b^CONAMA^39^.

**Figure 3:**
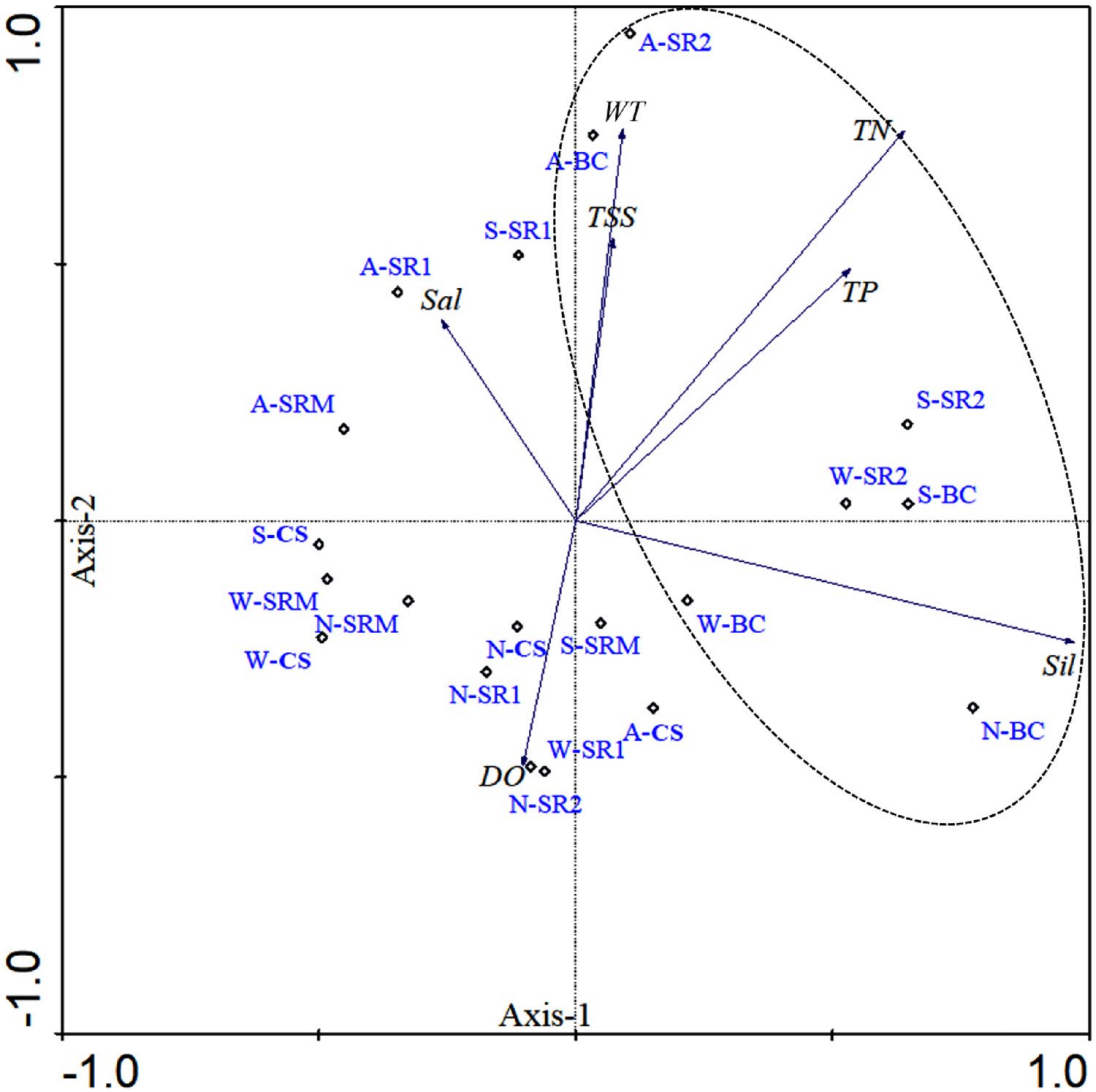
Principal compound analysis (PCA) for water quality parameters. Stations W-(BC, SR2, SR1, SRM, and CS) indicates the WM, A- (BC, SR2, SR1, SRM, and CS) indicates the SM, S-(BC, SR2, SR1, SRM, and CS) indicates the SWM, and N-(BC, SR2, SR1, SRM, and CS) indicates the NEM. Dotted circle indicates the upper estuarine stations.

The spatio-temporal distributions of environmental parameters were examined using principal component analysis (PCA) (Fig. 3). PCA result indicates a clear spatial variability in the study area. The first and second axis depicts 68.5% and 21.5% of the environmental variation in the SRE. The SR2 and BC stations were characterised by high WT (r^2^ = 0.63, *p* < 0.01), TSS (r^2^ = 0.67, *p* < 0.01) and nutrients (r^2^ = 0.58, *p* < 0.01). Among these environmental factors, SiO_4_^2–^, TN and TP were found to be the most influenced variables with higher values in the SR2 and BC stations. Hence, SR2 and BC stations were clearly distinguished by high nutrients, TSS attributed to anthropogenic runoff and mixing of sea & river water^14,35^.

### Spatio-temporal variations of phytoplankton community

During the present study area, phytoplankton biomass (Chl-*a*) varied from 0.17 to 17.18 mg m^−3^ between the seasons. SR2 and BC recorded higher biomass during the SWM and NEM, respectively (Table 1). Throughout the year about 5 to 6 times higher biomass was recorded at BC and SR2 compare to other stations (Table S1). Sewage-borne nutrients at BC and SR2 might have enhanced the phytoplankton biomass^42^. Low biomass was observed in the CS during the SM and SWM. A total of 81 phytoplankton species were identified in the present study, including 53 diatoms (32 centrales and 21 pennales), 24 dinoflagellates, 1 silicoflagellate, and 3 Cyanophycae (Table S2). The phytoplankton density varied from 5697 to 30,01,835 cells L^−1^, the mean density was higher during the SWM followed by WM and lower during the NEM and SM. Spatio-temporal variation of abundant phytoplankton species was presented in Fig. S1. Phytoplankton density was positively correlated with PO_4_^3–^ (r = 0.87, *p* < 0.01) and negatively correlated with pH (r = − 0.51, *p* < 0.05). During all the seasons, phytoplankton density was recorded maximum at BC station, which alone contributed 79–90% of total phytoplankton abundance, whereas in CS recorded only 1–5%. Spatially, the total phytoplankton density was recorded high in estuarine stations like BC (87%) followed by SR2 (7%) and low in SR1 (3%), CS (2%) and SRM (1%). In BC, 80–98% of the total abundance was contributed by a single species in each season, which shows the mono phytoplankton bloom or domination. In BC, the phytoplankton community was dominated by *Oscillatoria* sp. (96%), *Scrippsiella trochoidea* (87%), *Chaetoceros decipiens* (98%), and *Skeletonema costatum* (79%) during the WM, SM, SWM, and NEM, respectively. In all the 4 seasons, CS exhibited higher number of phytoplankton taxa and lower phytoplankton density, whereas in other estuarine stations high phytoplankton density and low taxa was observed. Higher nutrient concentrations by runoff and anthropogenic activities caused some phytoplankton species to dominate and resulting in low taxa and high density^19,43^. Diatoms found to be the most abundant group during the SWM and NEM, contributing 99% and 94%, respectively. Cyanophyceae (84%) and Dinophyceae (73%), dominated during the WM and SM, respectively. Phytoplankton density in BC station was high throughout the year (295.4 to 3001.8 × 10^3^ cells L^−1^). Diatoms showed positive correlation with PO_4_^3–^ (r = 0.92, *p* < 0.01), NO_2_^−^ (r = 0.62, *p* < 0.01), and negative correlation with pH (r = − 0.55, *p* < 0.05). Whereas the dinoflagellate resulted in negative correlation with nutrients, which reflected in lower abundance during all the seasons except the SM. Phytoplankton diversity ranged from 0.14 to 2.81, the higher diversity was found during the SWM at CS and lower diversity recorded during the WM at BC station. The species evenness ranged from 0.05 to 0.80 with higher and lower evenness observed during the SWM and NEM, respectively. During the SWM, the higher (3.47) and lower (0.74) species richness was observed at CS and BC, respectively. Throughout the season, the phytoplankton taxa was recorded high in CS (43) and low in estuarine region particularly at SR2 (10) and BC (12). In the CS, optimal hydrographic conditions and lower nutrient concentration lead to higher phytoplankton taxa and lower abundance, respectively. Whereas in estuarine stations, higher nutrient concentrations resulted in dominance of some phytoplankton species and higher TSS limiting the phytoplankton taxa^20,44,45^.

### Spatio-temporal distribution of dominant phytoplankton and HABs in relation to environmental variables

**Figure 4:**
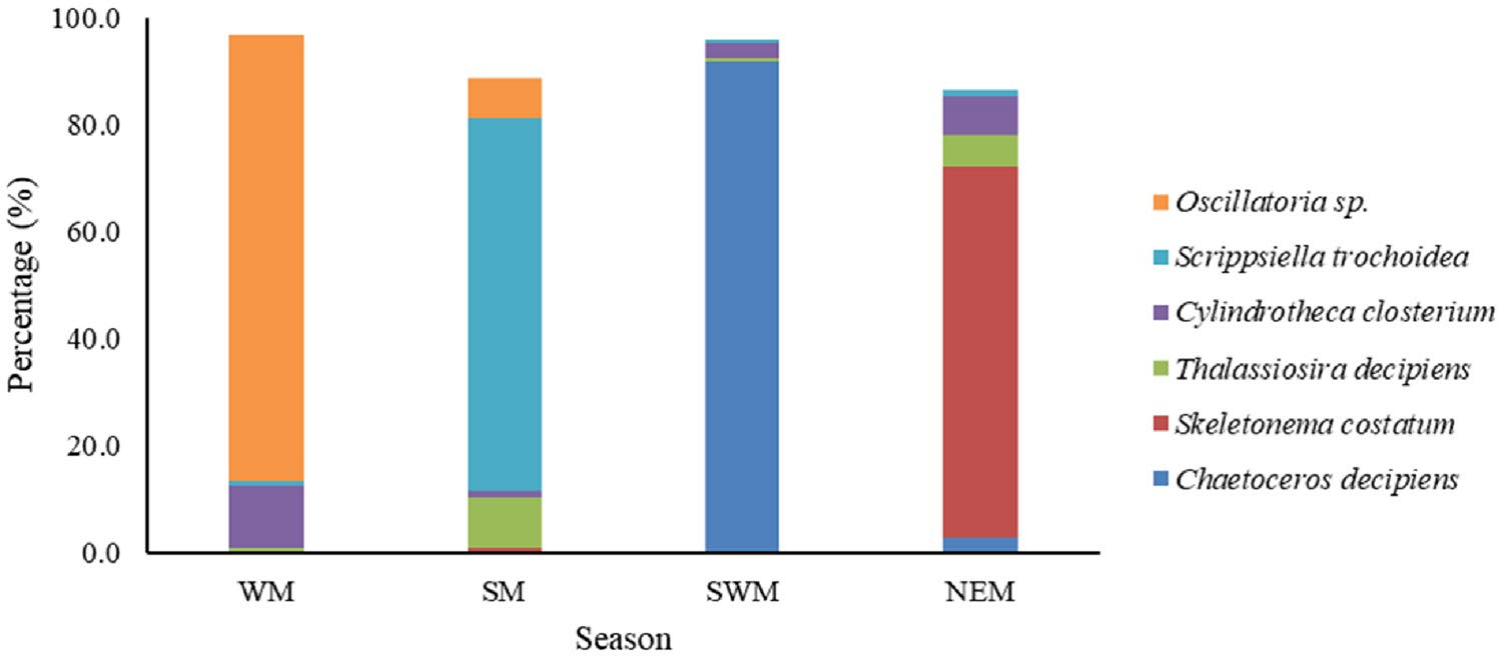
Seasonal distribution of most dominant phytoplankton species along the Swarnamukhi River Estuary, southeast coast of India.

**Figure 5:**
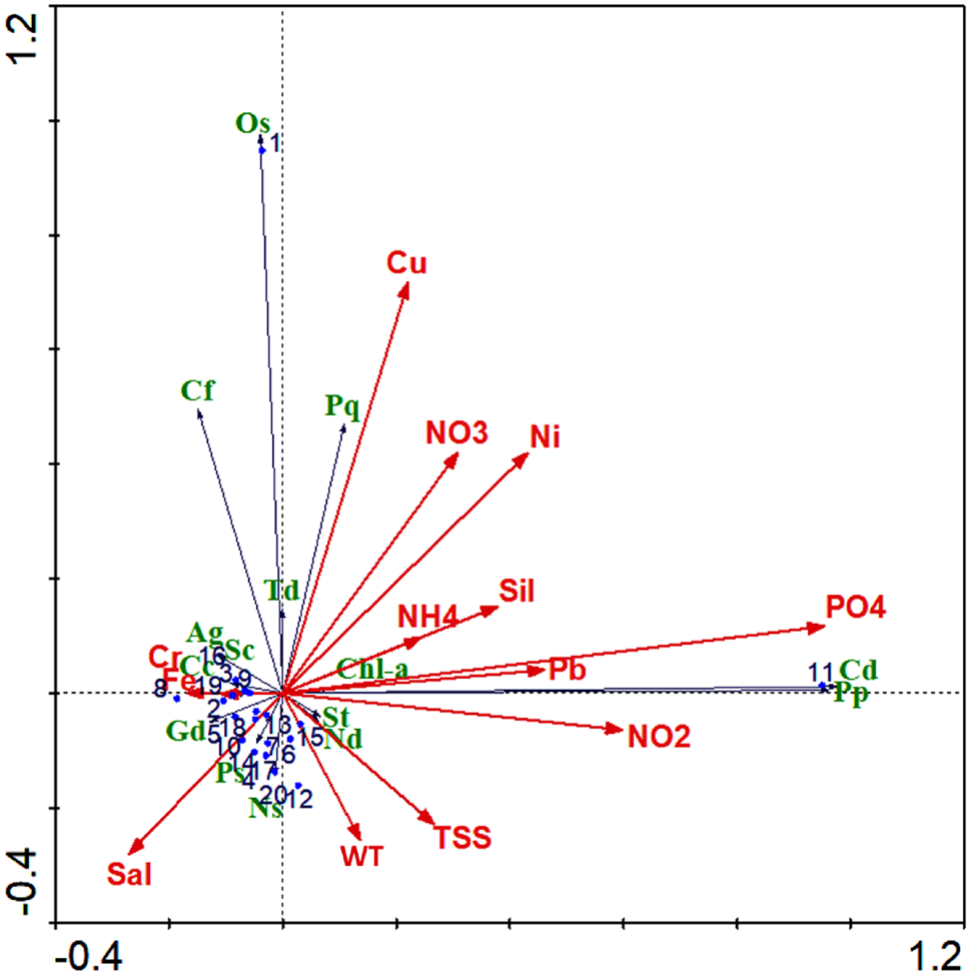
Redundancy analysis (RDA) for dominant phytoplankton species with associated environmental variables. The Chl-*a*, and phytoplankton species (Cd, *Chaetoceros decipiens*; Os, *Oscillatoria* sp.; Gd, *Guinardia delicatula*; Sc, *Skeletonema costatum*; Td, *Thalassiosira decipiens*; Ag, *Asterionellopsis glacialis*; Cc, *Cylindrotheca* c*losterium*; Nd, *Navicula delicatula*; Ps, *Pseudo-nitzschia* sp.; Cf, *Ceratium furca*; St, *Scrippsiella trochoidea*; Ns, *Noctiluca* sp.; Pq, *Peridinium quinquecorne* and Pp, *Protoperidinium pallidum*), stations {1–5 (BC, SR2, SR1, SRM, and CS of WM), 6–10 (BC, SR2, SR1, SRM, and CS of SM), 11–15 (BC, SR2, SR1, SRM, and CS of SWM), and 16–20 (BC, SR2, SR1, SRM, and CS of NEM)}, and environmental parameters {WT, water temperature; TSS, total suspended solids; Sal, salinity; NO2, nitrite; NO3, nitrate; NH4, ammonia; PO4, phosphate; Sil, silicate; Cu, copper; Ni, nickel; Pb, lead; Fe, iron and Cr, chromium}.

During the study period, 14 phytoplankton species (listed in Fig. 5) were found to be dominant, among these 6 species (*Chaetoceros decipiens*, *Oscillatoria* sp., *Scripsiella trochoidea*, *Skeletonema costatum*, *Cylindrotheca closterium*, and *Thalassiosira decipiens*) contributed 90–95% to the total phytoplankton abundance (Fig. 4). All these dominant phytoplankton species were observed at BC station except C. *closterium,* which was found at SR2. Throughout the seasons, the most abundant phytoplankton species were S. *trochoidea*, followed by C*. closterium* and T. *decipiens*. In BC, HABs of C. *decipiens* (2940 × 10^3^ cells L^−1^) and *Oscillatoria* sp. (1619 × 10^3^ cells L^−1^) occurred during the SWM (Jul 2019) and WM (Feb 2019), respectively. During the NEM, dominance of *Skeletonema costatum* (233.7 × 10^3^ cells L^−1^) was observed at BC might be attributed to higher silicate concentration (58.74 µM). Earlier study at Gurupura estuary also noted the bloom of *Skeletonema costatum* during the monsoon due to hefty silicate loading (> 100 µM)^46^. In comparison to other stations, BC was found to be very turbid and polluted, with high levels of TSS, heavy metals (Fe, Cu, Ni, Pb, Al, and Cd), and all the nutrients owing to agricultural runoff and frequent effluent discharge from shrimp farms. Earlier study also identified BC as a high-risk zone due to the various anthropogenic wastes such as fertilizers, algaecides, fungicides, and molluscicides in the vicinity^27^. Lack of riverine and oceanic influx due to low rainfall and the closure of the bar mouth, respectively resulted in the azoic condition (no species) at BC station^35^. Moderately high NH_4_^+^ concentration was found during the bloom period, which could be due to un-grazed and decomposition of the *Oscillatoria* sp. bloom^47,48^. Usually during the bloom of *Oscillatoria* sp., discolouration of water occurs and some instances causes mortality of fish, due to oxygen deficiency^49^. However, there were no fish kills sighted during the present study.

The redundancy analysis (RDA) was used to investigate the relationship between dominant phytoplankton species and environmental factors (Fig. 5). The physico-chemical variables in the first two axis explained 93.8% of the total variance of species distribution. The species-environmental factors in the first two axis explained 98.8% of the total variances with the eigenvalues of 0.720 and 0.217. In axis 1, *Chaetoceros decipiens* bloom was positively correlated with elevated nutrients (PO_4_^3–^, NO_2_^−^, and SiO_4_^2–^) and negatively correlated with salinity. During the C. *decipiens* bloom, higher WT (30.87 °C), PO_4_^3–^ (4.37 µM), and SiO_4_^2–^(50.35 µM) were recorded at BC. Earlier studies also revealed that *Chaetoceros* spp. bloom when the temperature, PO_4_^3–^, and SiO_4_^2–^ increases^50,51^. C. *decipiens* correlated positively with PO_4_^3–^ (r = 0.93, *p* < 0.01). Increased PO_4_^3–^ and SiO_4_^2–^ due to aquaculture and agriculture runoff might have triggered the bloom of C. *decipiens*. Chl-*a* was negatively correlated to salinity and positively correlated with PO_4_^3–^, Si, and NH_4_^+^. The majority of dinoflagellates and Cyanophyceae were positively correlated with nutrients and trace metals while being negatively correlated with salinity, WT, and TSS. In axis 2, bloom of *Oscillatoria* sp. was positively correlated to NO_3_^−^, Cu, Ni, and negatively correlated with salinity, where the high concentration of metals (Ni, Cu, and Cd) and low salinity were recorded at BC. Elevated Cd concentration observed in the bloom station could be attributed to the decomposition of detrital material produced by the *Oscillatoria* sp. bloom^52^. Rodriguez and Ho^53^ also reported that trace metal availability plays an important role in relieving the stress of *Oscillatoria* sp. induced by low red-light condition and promotes enhanced growth conditions. During the bloom period, higher Cu (4.24 ppb) and Ni (3.0 ppb) concentrations were observed in BC. The RDA results also revealed that Ni and Fe were positively correlated with *Oscillatoria* sp., which could have triggered bloom formation along with elevated nutrients at BC. Fluvial input is the main source for Ni in the estuarine region and it plays a vital role in algal growth^54–56^. Several studies examined the role of Ni and Fe in *Oscillatoria* growth^53,57–59^. Rodriguez and Ho^59^ found increased *Oscillatoria* growth in the presence of sufficient Ni concentration and high light, resulting in increased N_2_ fixation rates. Furthermore, the Cu concentration was high in BC, which is more sensitive to *Oscillatoria*^60,61^. Therefore, it can be concluded that high nutrients and presence of some heavy metals is playing a crucial role in phytoplankton productivity and bloom formation in the study region. Heavy metals are essential for phytoplankton growth at trace levels but, toxic at high levels^61^. Even though high Ni plays an important role in *Oscillatoria* sp. bloom increase in free Cu^2+^ concentration may be detrimental to *Oscillatoria* sp. growth^60,61^). However, more research is needed to determine the combination of certain metals in higher concentrations may have detrimental effect on the phytoplankton community and subsequent tropical level.

## Conclusion

In the present study, optimal hydrographic conditions and lower nutrient concentrations at coastal station was characterised by higher phytoplankton taxa and lower abundance, whereas in the estuarine region higher nutrient and trace metal concentrations from anthropogenic sources favoured the dominance of few phytoplankton species and lead to low phytoplankton diversity. Increased concentrations of NO_3_^−^ and Ni potentially triggered the *Oscillatoria* sp. bloom, whereas PO_4_^3–^ and SiO_4_^2–^ induced the bloom of *Chaetoceros decipiens*. Only the BC station documented the observed bloom episodes with the main cause as increased nutrient and metal content from aquaculture and other anthropogenic inputs. The present study will aid in anticipating their future response to climatic change, and it emphasizes the impact of trace metals in HABs, and future bloom events could harm zooplankton followed by fish and higher trophic level. Hence, treatment system and continuous monitoring are needed for the present study region.

### Supplementary Information

Supplementary Information.

## Data Availability

The datasets used and/or analyzed during the current study are available from the corresponding author on reasonable request.
