# Author Correction: Impact of anthropogenic accumulation on phytoplankton community and harmful algal bloom in temporarily open/closed estuary

**DOI:** 10.1038/s41598-024-53429-x

**Published:** 2024-02-05

**Authors:** Ponnusamy Sathish Kumar, Dharani Gopal, Dilip Kumar Jha, Krupa Ratnam, Santhanakumar Jayapal, Vikas Pandey, Venkatnarayanan Srinivas, Arthur James Rathinam

**Affiliations:** 1 Ocean Science and Technology for Islands, National Institute of Ocean Technology (NIOT), Ministry of Earth Sciences, Pallikaranai, Chennai, India; 2 Department of Marine Science, Bharathidasan University, Tiruchirappalli, Tamil Nadu India

Correction to: *Scientific Reports* 10.1038/s41598-023-47779-1, published online 27 December 2023

The original version of this Article contained an error in the name of the author Ponnusamy Sathish Kumar which was incorrectly given as Sathish Kumar Ponnusamy.

The original Article has been corrected.

