# Supplementary material for: Impact of anthropogenic accumulation on phytoplankton community and harmful algal bloom in temporarily open/closed estuary
Source: Sci Rep. 2023 Dec 27;13:23034. doi: 10.1038/s41598-023-47779-1 (PMC10754910; doi:10.1038/s41598-023-47779-1)
Supplement: Supplementary file 1 — Supplementary Information. [file 41598_2023_47779_MOESM1_ESM.docx]

**Supplementary Figure**

**Fig. S1** Spatio-temporal distribution of abundant phytoplankton species along the Swarnamukhi River Estuary, southeast coast of India (Scale: Log (x+1) transformed)


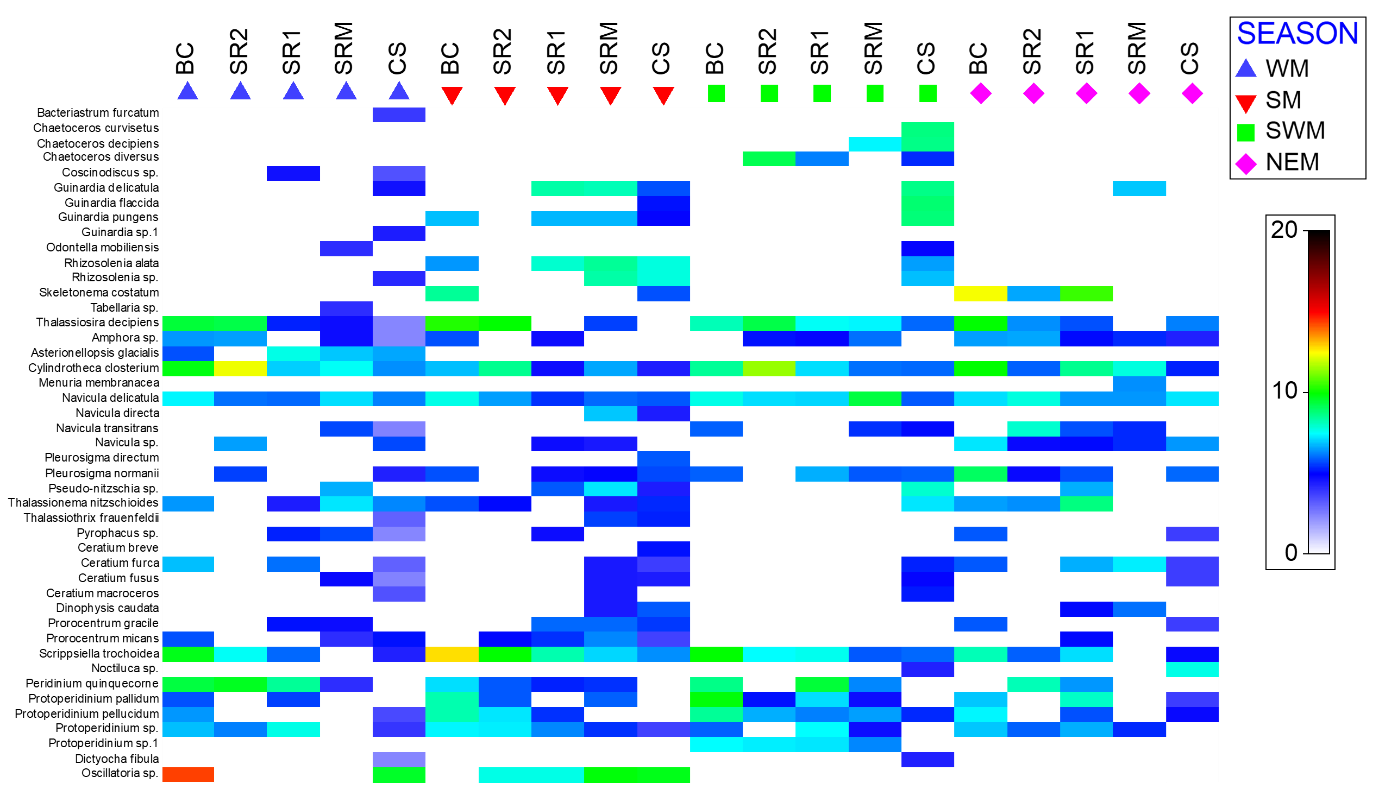


**Supplementary Tables**

**Table S1.** Spatial variation of environmental variables in the Swarnamukhi River Estuary, southeast coast of India. Values in the open and parentheses represent the minimum - maximum and mean values with ± standard error respectively.

| **Parameters** | **BC** | **SR2** | **SR1** | **SRM** | **CS** |
| --- | --- | --- | --- | --- | --- |
| SST (°C) | 28.02-32.77 | 28.75-32.93 | 27.95-32.70 | 27.39-31.59 | 26.64-30.67 |
|  | (30.17±1.05) | (30.33±0.96) | (30.13±1.05) | (29.81±0.88) | (29.23±0.89) |
|  |  |  |  |  |  |
| pH | 8.06-8.27 | 8.18-8.39 | 8.14-8.40 | 8.23-8.37 | 8.21-8.33 |
|  | (8.18±0.05) | (8.30±0.05) | (8.26±0.05) | (8.33±0.03) | (8.28±0.03) |
| Salinity (PSU) | 28.0-33.27 | 28.93-32.46 | 27.71-33.76 | 28.54-32.89 | 31.40-33.31 |
|  | (29.95±1.24) | (29.94±0.84) | (29.57±1.41) | (30.32±1.03) | (32.11±0.42) |
|  |  |  |  |  |  |
| TSS (mg L^-1^) | 8.60-19.50 | 5.30-16.50 | 6.53-16.30 | 14.05-20.80 | 5.87-14.06 |
|  | (15.45±2.42) | (11.45±2.72) | (12.26±2.11) | (16.80±1.51) | (9.76±1.95) |
| DO (mg L^-1^) | 2.28 - 4.15 | 3.74 - 5.12 | 3.17 - 4.39 | 3.01 - 5.12 | 3.82 - 5.20 |
|  | (3.50±0.42) | (4.25±0.31) | (3.76±0.25) | (4.07±0.43) | (4.70±0.32) |
| BOD (mg L^-1^) | 0.0 - 3.58 | 0.0 - 4.23 | 1.05 - 3.74 | 1.95 - 4.15 | 1.41 - 3.10 |
|  | (2.26±0.80) | (2.84±0.97) | (2.45±0.58) | (2.82±0.49) | (2.33±0.45) |
| NH_4_^+^ (µM) | 0.1-7.25 | 0.1-7.44 | 0.1-6.73 | 0.40-9.33 | 0.73-1.05 |
|  | (4.41±1.60) | (2.99±1.59) | (2.37±1.49) | (4.79±2.34) | (0.94±0.07) |
|  |  |  |  |  |  |
| NO_3_^−^ (µM) | 0.31-10.18 | 2.14-11.23 | 0.48-5.63 | 0.70-3.78 | 0.53-3.06 |
|  | (7.27±2.34) | (5.71±2.18) | (3.90±1.16) | (2.25±0.63) | (1.58±0.54) |
|  |  |  |  |  |  |
| NO_2_^−^ (µM) | 0.12-2.10 | 0.23-1.63 | 0.17-0.76 | 0.31-0.43 | 0.13-0.22 |
|  | (1.06±0.48) | (0.94±0.39) | (0.42±0.12) | (0.35±0.03) | (0.17±0.02) |
|  |  |  |  |  |  |
| PO_4_^3−^ (µM) | 0.21-4.37 | 0.08-1.03 | 0.10-1.14 | 0.19-0.49 | 0.36-1.18 |
|  | (1.62±0.93) | (0.41±0.21) | (0.44±0.24) | (0.39±0.07) | (0.64±0.18) |
|  |  |  |  |  |  |
| SiO_4_^2−^ (µM) | 21.35-58.74 | 21.64-48.50 | 9.92-28.25 | 8.58-30.59 | 8.69-35.32 |
|  | (41.44±8.27) | (35.54±6.57) | (19.63±3.91) | (16.55±5.01) | (19.63±6.36) |
|  |  |  |  |  |  |
| TN (µM) | 17.69-30.35 | 6.24-37.12 | 6.33-24.23 | 4.44-11.57 | 5.09-11.79 |
|  | (23.47±2.84) | (24.98±6.61) | (14.08±4.43) | (8.06±1.97) | (8.13±1.47) |
|  |  |  |  |  |  |
| TP (µM) | 0.21-4.37 | 0.08-1.03 | 0.10-1.14 | 0.19-0.49 | 0.36-1.18 |
|  | (1.62±0.93) | (0.41±0.21) | (0.44±0.24) | (0.39±0.07) | (0.64±0.18) |
|  |  |  |  |  |  |
| Chl-*a* (mg m^-3^) | 4.53-7.95 | 1.13-17.18 | 0.44-2.39 | 0.35-0.80 | 0.17-2.15 |
|  | (6.21±0.93) | (5.78±3.82) | (1.46±0.43) | (0.51±0.10) | (0.69±0.49) |
| Phytoplankton Density  (×10^3^ Cells L^-1^) | 295.4-3001.8 | 12.4-237.2 | 12.2-120.0 | 7.5-40.2 | 5.7-49.7 |
|  | (1340.6±638.2) | (111.0±49.9) | (50.7±25.0) | (21.1±6.9) | (24.6±9.3) |
|  |  |  |  |  |  |

**Table S2.** Phytoplankton species composition along the Swarnamukhi River Estuary, southeast coast of India. (–) absent, (+) 1–100, (++) 101–1000, (+++) 1001–10000, and (++++) above 10000 Cells L^–1^

| **Station** | **WM** | **SM** | **SWM** | **NEM** |
| --- | --- | --- | --- | --- |
| **Diatoms (centrics)** |  |  |  |  |
| *Asterolampra* sp. | + | - | - | - |
| *Bacteriastrum furcatum* | + | - | - | - |
| *Bellerochea* sp. | - | + | ++ | - |
| *Chaetoceros curvisetus* | - | - | +++ | - |
| *Chaetoceros decipiens* | - | - | ++++ | +++ |
| *Chaetoceros diversus* | - | - | +++ | - |
| *Coscinodiscus centralis* | + | - | + | - |
| *Coscinodiscus* sp. | + | - | - | - |
| *Cyclotella* sp. | - | - | - | ++ |
| *Ditylum* sp. | - | - | + | - |
| *Eucampia zodiacus* | - | - | ++ | - |
| *Guinardia delicatula* | + | +++ | +++ | ++ |
| *Guinardia flaccida* | - | + | +++ | - |
| *Guinardia pungens* | - | ++ | +++ | - |
| *Guinardia striata* | - | - | + | - |
| *Guinardia* sp.1 | + | - | - | - |
| *Hemiaulus* sp. | - | - | + | - |
| *Hemidiscus sp.* | - | - | + | + |
| *Leptocylindrus* sp. | - | - | ++ | - |
| *Melosira* sp. | - | - | ++ | - |
| *Odontella mobiliensis* | + | - | + | - |
| *Odontella rhombus* | + | - | - | - |
| *Odontella sinensis* | - | - | + | - |
| *Odontella* sp. | - | + | - | - |
| *Rhizosolenia alata* | - | +++ | ++ | - |
| *Rhizosolenia imbricata* | - | - | ++ | - |
| *Rhizosolenia* sp.1 | - | - | + | - |
| *Rhizosolenia sp.2* | + | +++ | ++ | - |
| *Skeletonema costatum* | - | ++ | - | ++++ |
| *Tabellaria* sp. | + | - | - | - |
| *Thalassiosira decipiens* | +++ | +++ | +++ | +++ |
| *Triceratium* sp. | - | - | + | - |
| **Diatoms (pennates)** |  |  |  |  |
| *Amphora* sp. | ++ | + | ++ | ++ |
| *Asterionellopsis glacialis* | ++ | - | - | - |
| *Cylindrotheca closterium* | ++++ | +++ | ++++ | +++ |
| *Diploneis* sp. | + | + | + | - |
| *Licmophora* sp. | + | - | - | - |
| *Menuria membranacea* | - | - | - | ++ |
| *Navicula delicatula* | ++ | ++ | +++ | +++ |
| *Navicula directa* | - | ++ | - | - |
| *Navicula distans* | - | + | + | - |
| *Navicula transitrans* | + | - | ++ | ++ |
| *Navicula* sp. | ++ | + | - | ++ |
| *Nitzschia longissima* | + | - | - | - |
| *Nitzschia sigma* | + | - | + | - |
| *Pleurosigma angulatum* | ++ | + | + | + |
| *Pleurosigma directum* | - | + | - | - |
| *Pleurosigma normanii* | + | ++ | ++ | +++ |
| *Pseudo-nitzschia* sp. | ++ | ++ | ++ | ++ |
| *Surirella* sp. | + | - | - | - |
| *Thalassionema nitzschioides* | ++ | ++ | ++ | +++ |
| *Thalassiothrix frauenfeldii* | + | + | - | - |
| *Thalassiothrix* sp. | - | - | + | + |
| **Dinoflagellates** |  |  |  | - |
| **Autotrophic** |  |  |  |  |
| *Gymnodinium* sp. | ++ | + | - | - |
| *Pyrophacus* sp. | + | + | - | + |
| **Mixotrophic** |  |  |  |  |
| *Ceratium breve* | - | + | - | - |
| *Ceratium furca* | ++ | + | + | ++ |
| *Ceratium fusus* | + | + | + | + |
| *Ceratium macroceros* | + | + | + | - |
| *Ceratium tripos* | + | + | - | - |
| *Dinophysis caudata* | - | + | - | ++ |
| *Prorocentrum gracile* | + | ++ | - | + |
| *Prorocentrum lima* | + | - | - | + |
| *Prorocentrum micans* | ++ | ++ | - | + |
| *Scrippsiella trochoidea* | +++ | ++++ | +++ | +++ |
| **Heterotrophic** |  |  |  |  |
| *Gyrodinium* sp. | ++ | - | - | + |
| *Noctiluca* sp. | - | - | + | ++ |
| *Ornithocercus thumii* | + | - | - | - |
| *Peridinium quinquecorne* | +++ | ++ | +++ | ++ |
| *Protoperidinium curtipes* | + | - | - | - |
| *Protoperidinium depressum* | - | + | - | - |
| *Protoperidinium divergens* | - | ++ | + | - |
| *Protoperidinium pallidum* | ++ | ++ | +++ | ++ |
| *Protoperidinium pellucidum* | ++ | +++ | +++ | ++ |
| *Protoperidinium* sp.1 | - | - | - | ++ |
| *Protoperidinium* sp.2 | ++ | ++ | ++ | ++ |
| *Protoperidinium* sp.3 | - | - | +++ | - |
| **Silicoflagellates** |  |  |  | - |
| *Dictyocha fibula* | + | - | + | - |
| **Cyanophyceae** |  |  |  | - |
| *Oscillatoria* sp. | ++++ | +++ | - | - |
| *Cyanophyceae UK-1* | - | - | ++ | - |
| *Cyanophyceae UK-2* | ++ | - | - | - |
